# Application of thromboelastogram and coagulation function in evaluating coagulation status of pregnant women across different trimesters

**DOI:** 10.3389/fmed.2025.1711912

**Published:** 2025-12-12

**Authors:** Jiayu Li, Jianfen Zhu, Xiaoqian Chen, Mingyu Wang, Ying Sha

**Affiliations:** 1 Clinical Laboratory, Zhangjiagang Second People’s Hospital, Zhangjiagang, Jiangsu, China; 2 Department of Obstetrics and Gynecology, Zhangjiagang Second People’s Hospital, Zhangjiagang, Jiangsu, China

**Keywords:** coagulation function, perinatal outcomes, pregnancy, thromboelastogram, trimesters

## Abstract

**Background:**

Pregnancy induces a hypercoagulable state to prevent hemorrhage during delivery, but it also increases the risk of thrombotic events and postpartum hemorrhage. Monitoring coagulation changes is essential to ensure maternal and fetal health. Thromboelastography (TEG), which provides a detailed assessment of the coagulation cascade, remains underutilized across all stages of pregnancy.

**Methods:**

This retrospective study included 92 pregnant women grouped by trimester (30 in the first, 34 in the second, and 28 in the third), and 30 non-pregnant women as controls. Standard coagulation metrics [prothrombin time (PT), activated partial thromboplastin time (APTT), thrombin time (TT) and fibrinogen (FIB)] and TEG parameters (R, K, *α*-angle, MA) were measured and compared.

**Results:**

As pregnancy progressed, PT, APTT, and TT decreased, while Fibrinogen levels increased significantly compared to the control group (*p* < 0.05). TEG results showed a decrease in R and K values, with an increase in *α*-angle and MA across trimesters (*p* < 0.05). ROC analysis showed that combining TEG with traditional tests improved the accuracy of assessing coagulation status, yielding an AUC of 0.876, sensitivity of 84.0%, and specificity of 83.6%.

**Conclusion:**

Significant changes in both TEG and traditional coagulation parameters throughout pregnancy reflect the development of a hypercoagulable state. The integration of TEG with standard tests offers a comprehensive approach for detecting coagulation abnormalities early, improving perinatal outcomes. These findings are descriptive of physiological adaptation and do not support routine TEG screening in healthy pregnancies.

## 1 Introduction

The coagulation and fibrinolytic systems maintain a dynamic balance through mutual regulation in healthy individuals. When the body is stimulated by endogenous or exogenous factors, the coagulation cascade becomes activated, leading to the activation of previously inactive coagulation factors, ultimately resulting in coagulation (1). Pregnancy is an internal physiological stimulus that affects the balance between coagulation and fibrinolysis. As pregnancy advances, the balance between coagulation and fibrinolysis shifts toward a physiological hypercoagulable state (2). The normal physiological hypercoagulable state of pregnancy is beneficial because it facilitates rapid hemostasis at delivery and reduces the risk of postpartum hemorrhage. However, an abnormal hypercoagulable state may be associated with complications such as preeclampsia and gestational diabetes mellitus, thereby increasing the risk of adverse perinatal outcomes including preterm delivery and postpartum hemorrhage (3, 4). Therefore, it is crucial to monitor coagulation in pregnant women at different stages of gestation to optimize pregnancy management. Traditional coagulation tests, including PT, APTT, TT, and FIB, are effective tools for evaluating coagulation function. However, due to the unique nature of pregnancy, the coagulation function of pregnant women differs significantly from that of non-pregnant women, with increased coagulation activity during pregnancy (5, 6). Therefore, relying solely on these four plasma-based indices cannot fully reflect the overall coagulation status of pregnant women. TEG has shown promise in surgical, oncological, and emergency settings, as well as in patients receiving anticoagulant therapy (7, 8). By analyzing whole blood samples, TEG can dynamically depict the process from coagulation initiation to fibrin formation, cross-linking, and dissolution (2, 9). Thakkar et al. (10) suggested that TEG can assist in detecting pathological changes in coagulation, facilitate transfusion management and targeted anticoagulation therapy, and help predict adverse events, thereby potentially reducing morbidity. Although TEG and other viscoelastic assays have been evaluated in specific obstetric contexts—including women with gestational hypertension, preeclampsia, or postpartum hemorrhage—trimester-stratified data in strictly defined healthy, low-risk pregnant women remain relatively limited, particularly regarding the combined interpretation of TEG indices and conventional plasma-based coagulation tests. Therefore, in the present study we focused on healthy pregnant women at different stages of gestation to characterize physiological, trimester-specific changes in both conventional coagulation tests and TEG parameters and to explore whether their combination improves the laboratory assessment of hypercoagulability. Our aim was to provide physiological reference information that may help contextualize TEG findings where viscoelastic testing is already employed, rather than to advocate for routine, population-level TEG screening in all pregnant women.

## 2 Materials and methods

### 2.1 Subjects

Because TEG can assess overall hemostatic function and is sensitive to coagulopathy, it was used in this retrospective study to evaluate pregnant women with a focus on coagulation status. We conducted a retrospective study of pregnant women managed in the Department of Obstetrics and Gynecology at Zhangjiagang Second People’s Hospital from April 2022 to October 2023.

Inclusion criteria for this study were as follows:

1. Singleton pregnancy conceived naturally.
2. Normal blood pressure without proteinuria or pregnancy-related complications.
3. Delivery through vaginal birth.
4. Gestational age between 37 and 42 weeks at delivery.
5. Complete clinical data available, including TEG and coagulation function tests.

Exclusion criteria for this study were as follows:

1. Use of medications affecting blood coagulation during pregnancy.
2. History of hematological diseases or coagulation abnormalities.
3. History of blood transfusion or major surgery.
4. History of abnormal abortion or abnormal fetal development.
5. Patients with cardiovascular and cerebrovascular diseases.
6. Patients with pregnancy complications such as gestational hypertension, diabetes, hyperthyroidism, hypothyroidism, etc.
7. Abnormal placental function.
8. Patients with mental illness.

### 2.2 Blood collection timing and sample processing protocols

For pregnant women, blood samples were collected between 7:00 and 9:00 a.m. after an overnight fast to minimize diurnal variation. Samples were obtained according to gestational age: in the first trimester (<12 weeks) during routine early prenatal screening; in the second trimester (13–28 weeks) during mid-pregnancy check-ups; and in the third trimester (>28 weeks) during pre-delivery evaluation. For the non-pregnant control group, blood collection was scheduled under similar fasting conditions.

Immediately following collection, whole blood samples were divided into two aliquots. One aliquot was processed for standard coagulation tests (PT, APTT, TT, and fibrinogen) using an automated coagulation analyzer within 2 h of collection. The second aliquot was used for TEG analysis. Samples designated for TEG were maintained at room temperature and were analyzed within 30 min of collection using the TEG 5000 analyzer. Standard operating procedures for TEG included daily calibration checks using manufacturer-provided quality control materials to ensure measurement consistency.

### 2.3 Quality control measures for TEG

For each TEG run, we performed internal quality control by running standard reference samples, and the TEG machine calibration was verified with manufacturer-provided controls. Additionally, each TEG parameter (R, K, *α*-angle, and MA) was measured in duplicate, and the average value was used for further analysis. Any discrepancy greater than 5% between duplicates triggered repeat testing.

R (reaction time): An R value below 5 min indicates rapid clot initiation, suggestive of hypercoagulability.

K (clot kinetics): A K value less than 1 min suggests faster clot formation, indicative of enhanced thrombin generation.

*α*-angle: An α-angle greater than 72° demonstrates increased fibrin formation and stronger clot structure.

MA (maximum amplitude): An MA value over 70 mm indicates high clot strength, primarily due to increased platelet function or fibrinogen levels.

### 2.4 Handling of missing data

Any missing or incomplete data were verified against the original medical records. If any variable for a subject was missing, that subject was excluded from the specific analysis related to the missing parameter. However, no subject had more than 5% of the total data missing, and missing values did not exceed 2% per variable. The pattern of missingness was investigated, and no systematic bias was identified.

Power calculations were conducted to justify the sample size. Based on an *α* of 0.05, a one-tailed *β* of 0.14 (power = 0.86), the required sample size was calculated as 92 participants using the following formula:

The sample size formula:


(μa+μβ)2σ2δ2+14μα2

**Figure 1:**
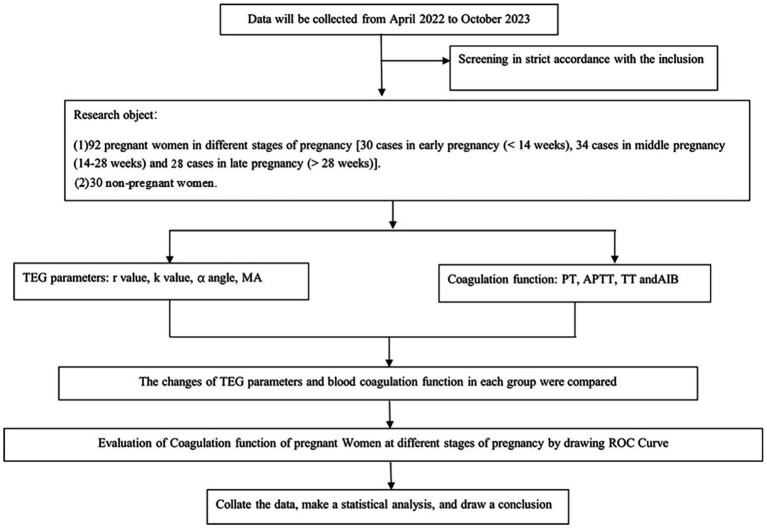
Research flow chart. PT, prothrombin time; APTT, activated partial thromboplastin time; TT, thrombin time; FIB, fibrinogen; TEG, thromboelastography; R, reaction time.

Hence, a total of 92 pregnant women were enrolled to ensure sufficient statistical power to detect significant differences. According to the classification standard of pregnancy (11), pregnant women were assigned to groups based on the trimester at which their laboratory tests were recorded. Specifically, 30 cases were in the first trimester (<12 weeks), 34 in the second trimester (13–28 weeks), and 28 in the third trimester (>28 weeks). No patient had repeated laboratory tests in subsequent trimesters or in the postpartum period. Age, systolic blood pressure, diastolic blood pressure, and fasting blood glucose did not differ significantly among the four groups (*p* > 0.05), as summarized in Table 1. Group-wise PT, APTT, TT, and FIB values are presented separately in Table 2. During the same period, 30 non-pregnant women who underwent routine health examinations at our hospital were selected as the control group. None of these individuals had heart, liver, kidney, blood system, or autoimmune system diseases, and none were using medications affecting blood coagulation. The specific research flow chart is shown in Figure 1.

**Table 1:** General clinical characteristics of each group of subjects.

Group	Age (years)	Systolic blood pressure (mmHg)	Diastolic blood pressure (mmHg)	Fasting blood glucose (mmol/L)
Control group (*n* = 30)	25.95 (24.37, 28.74)	114.51 (112.45, 117.99)	86.26 (84.89, 88.08)	4.03 (3.87, 4.32)
First trimester group (*n* = 30)	26.38 (24.28, 28.84)	116.56 (113.45, 121.69)	87.25 (83.18, 89.85)	4.04 (3.80, 4.41)
Second trimester group (*n* = 34)	27.89 (26.39, 28.77)	115.19 (111.05, 119.31)	86.49 (85.28, 87.23)	4.15 (3.81, 4.45)
Third trimester group (*n* = 28)	25.52 (23.82, 27.63)	115.32 (113.94, 118.60)	86.80 (85.21, 87.65)	4.21 (3.93, 4.49)
H	7.275	3.427	5.812	1.666
*p*	0.051	0.330	0.281	0.645

H = Kruskal–Wallis H test statistic.

**Table 2:** Comparison of four items of coagulation function of pregnant women in different periods of pregnancy.

Group	PT (S)	APTT (S)	TT (S)	FIB (g/L)
Control group (*n* = 30)	10.65 (8.89, 13.81)	30.18 (25.85, 34.15)	15.85 (14.10, 17.85)	2.72 (2.25, 3.32)
First trimester group (*n* = 30)	8.46 (7.92, 11.42)^a^	26.39 (20.49, 29.47)^a^	14.13 (13.84, 18.36)^a^	3.34 (2.98, 3.46)^a^
Second trimester group (*n* = 34)	8.11 (6.54, 9.34)^ab^	24.97 (22.27, 27.13)^ab^	13.52 (12.26, 14.60)^ab^	4.05 (3.56, 4.52)^ab^
Third trimester group (*n* = 28)	7.98 (5.68, 9.58)^abc^	22.41 (17.38, 27.14)^abc^	10.89 (9.34, 11.92)^abc^	4.80 (4.35, 5.43)^abc^
*H*	23.636	17.870	46.430	81.948
*p*	<0.001	<0.001	<0.001	<0.001

Compared with the control group, ^a^*p* < 0.05; compared with the first trimester group, ^b^*p* < 0.05; compared with the second trimester group, ^c^*p* < 0.05. H = Kruskal–Wallis H test statistic; PT, prothrombin time; APTT, activated partial thromboplastin time; TT, thrombin time; FIB, fibrinogen.

### 2.5 Research contents and methods

Specific details such as general data collection, collection of research specimens, traditional blood coagulation tests, and TEG detection were provided in the Supplementary material.

### 2.6 Outcome measures

1. Comparison of conventional coagulation indices among pregnant women at different stages of pregnancy.
2. Comparison of TEG parameters among pregnant women at different stages of pregnancy.
3. TEG and conventional coagulation indices were jointly used to evaluate coagulation status across gestation. Abnormal hypercoagulability was defined *a priori* based on our laboratory reference intervals, informed by obstetric viscoelastic-testing literature: PT < 9 s, APTT < 26 s, TT < 15 s, and fibrinogen (FIB) > 4 g/L. For TEG, abnormality thresholds were R < 5 min, K < 1 min, *α*-angle > 72°, and MA > 70 mm. The occurrence of abnormal hypercoagulability served as the state variable in subsequent ROC analyses (5, 7, 8, 12).

### 2.7 Statistical analysis

SPSS 22.0 was utilized for statistical analysis, and GraphPad Prism 9 was used for data visualization. The Kolmogorov–Smirnov test was employed to assess data distribution. Measurement data showing non-normal distribution were presented as median (minimum-maximum), and group comparisons were performed using the non-parametric Kruskal–Wallis test. Categorical variables were expressed as number (percentage), and between-group comparisons were made using the *χ*^2^ test. Receiver operating characteristic (ROC) curves were constructed to evaluate the ability of TEG and conventional coagulation tests to identify abnormal hypercoagulability in pregnant women at different stages of gestation. The analysis utilized the Area Under the Curve (AUC) metric, where differences with a *p*-value less than 0.05 were deemed statistically significant.

For the combined model, all eight variables (PT, APTT, TT, FIB, R, K, *α*-angle, MA) were *z*-score standardized and entered into a multivariable logistic regression to estimate the probability of abnormal hypercoagulability. ROC curves and AUCs were obtained for each single marker and for the combined model. The optimal cut-off was chosen by the Youden index; corresponding sensitivity and specificity are reported. *p*-values for AUCs refer to the null hypothesis AUC = 0.5.

## 3 Results

### 3.1 Comparison of general data of each group of subjects

There was no significant difference in the general clinical characteristics among the four groups, including the control group (*p* > 0.05), as shown in Table 1.

### 3.2 Comparison of four parameters of coagulation function in different stages of pregnancy

**Figure 2:**
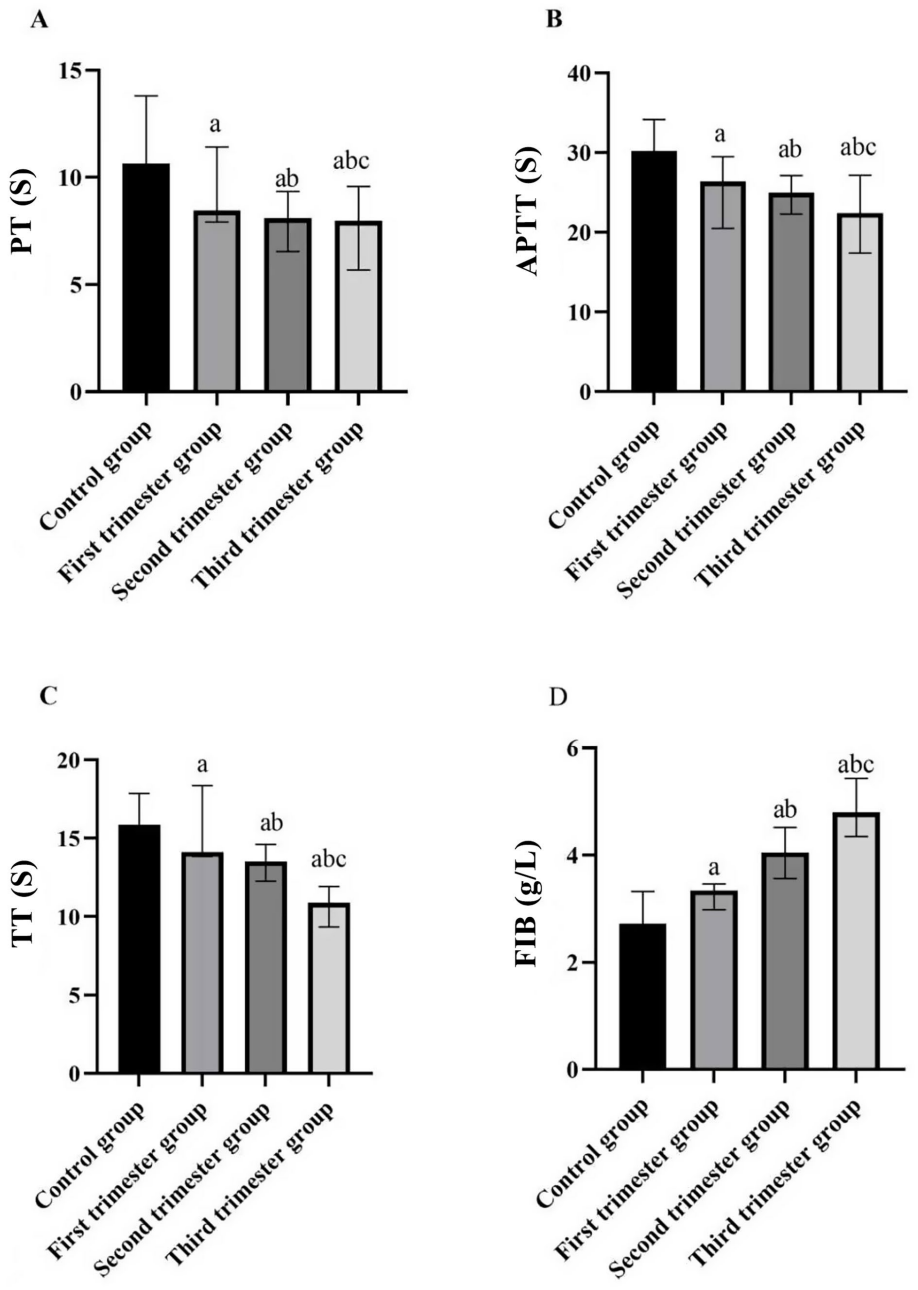
Comparison of four indices of blood coagulation function in different stages of pregnancy and the control group. **(A)** PT (s); **(B)** APTT (s); **(C)** TT (s); **(D)** FIB (g/L). (compared with the control group, ^a^*p* < 0.05; compared with the first trimester group, ^b^*p* < 0.05; compared with the second trimester group, ^c^*p* < 0.05). PT: prothrombin time; APTT: activated partial thromboplastin time; TT: thrombin time; FIB: fibrinogen.

Across the stages of pregnancy, prothrombin time (PT), activated partial thromboplastin time (APTT), and thrombin time (TT) showed a decreasing trend, whereas fibrinogen (FIB) levels increased as gestational age advanced (*p* < 0.05). More specifically, when compared with the control group, the values of PT, APTT, and TT showed a significant decrease in the first trimester group, the second trimester group, and the second trimester group (*p* < 0.05). Concurrently, FIB levels exhibited an increase across these stages (*p* < 0.05), as depicted in Table 2 and Figure 2.

### 3.3 Comparison of TEG of pregnant women in different periods of pregnancy

**Figure 3:**
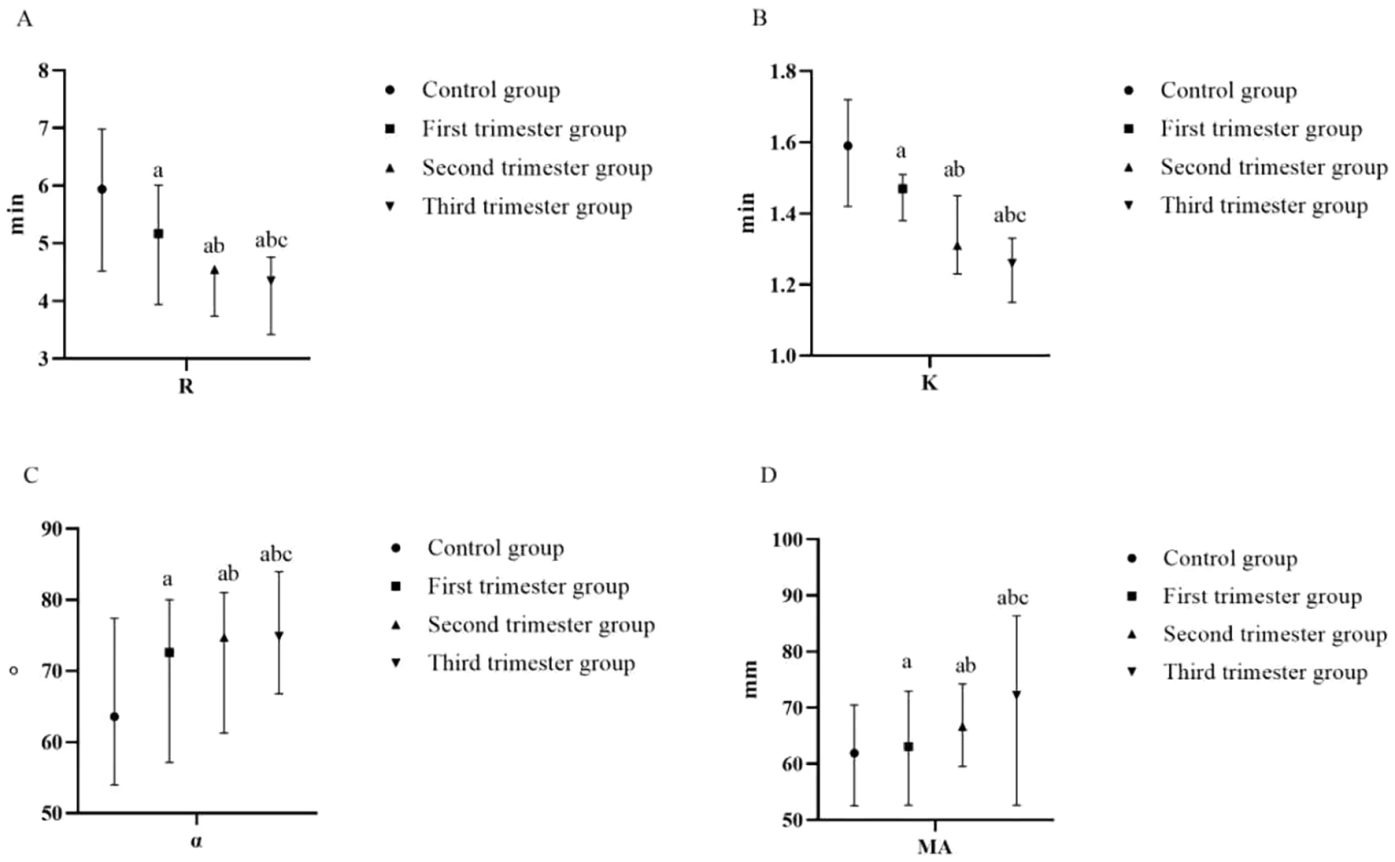
Comparison of TEG of pregnant women at different stages of pregnancy and the control group **(A)** R (min); **(B)** K (min); **(C)** α-angle (°); **(D)** MA (mm). (compared with the control group, ^a^*p* < 0.05; compared with the first trimester group, ^b^*p* < 0.05; compared with the second trimester group, ^c^*p* < 0.05). R, reaction time; K, kinetics time; *α*-angle, angle of clot formation; MA, maximum amplitude.

With the prolongation of gestational weeks, the values of R and K decreased, while the values of *α* angle and MA increased (*p* < 0.05). Compared with the control group, the values of R and K in the first trimester group, the second trimester group and the second trimester group decreased, while the values of α angle and MA increased (*p* < 0.05), as shown in Table 3 and Figure 3.

**Table 3:** Comparison of TEG of pregnant women in different periods of pregnancy.

Group	R value (min)	K value (min)	α Angle (°)	MA VALUE (mm)
Control group (*n* = 30)	5.94 (4.52, 6.98)	1.59 (1.41, 1.72)	63.57 (53.98, 77.42)	61.88 (52.53, 70.47)
First trimester group (*n* = 30)	5.17 (3.94, 6.01)^a^	1.47 (1.38, 1.51)^a^	72.62 (57.15, 80.02)^a^	63.07 (52.59, 72.94)^a^
Second trimester group (*n* = 34)	4.55 (3.74, 4.55)^ab^	1.31 (1.23, 1.45)^ab^	74.76 (61.29, 81.00)^ab^	66.63 (59.54, 74.21)^ab^
Third trimester group (*n* = 28)	4.35 (3.42, 4.76)^abc^	1.26 (1.15, 1.33)^abc^	74.88 (66.78, 83.95)	72.19 (52.60, 86.35)^abc^
*H*	15.474	51.606	12.941	10.426
*p*	<0.001	<0.001	<0.001	<0.001

Compared with the control group, ^a^*p* < 0.05; compared with the first trimester group, ^b^*p* < 0.05; compared with the second trimester group, ^c^*p* < 0.05. R = Reaction time, representing the latency period until initial fibrin formation (min); K = Clot formation time, indicating the speed of clot strengthening (min); α-angle = Angle between the baseline and the tangent to the clotting curve, reflecting the rate of fibrin build-up (°); MA = Maximum amplitude, representing the maximum strength of the formed clot (mm); H = Kruskal–Wallis H test statistic.

### 3.4 The value of TEG and coagulation function in evaluating coagulation function of pregnant women in different periods

**Figure 4:**
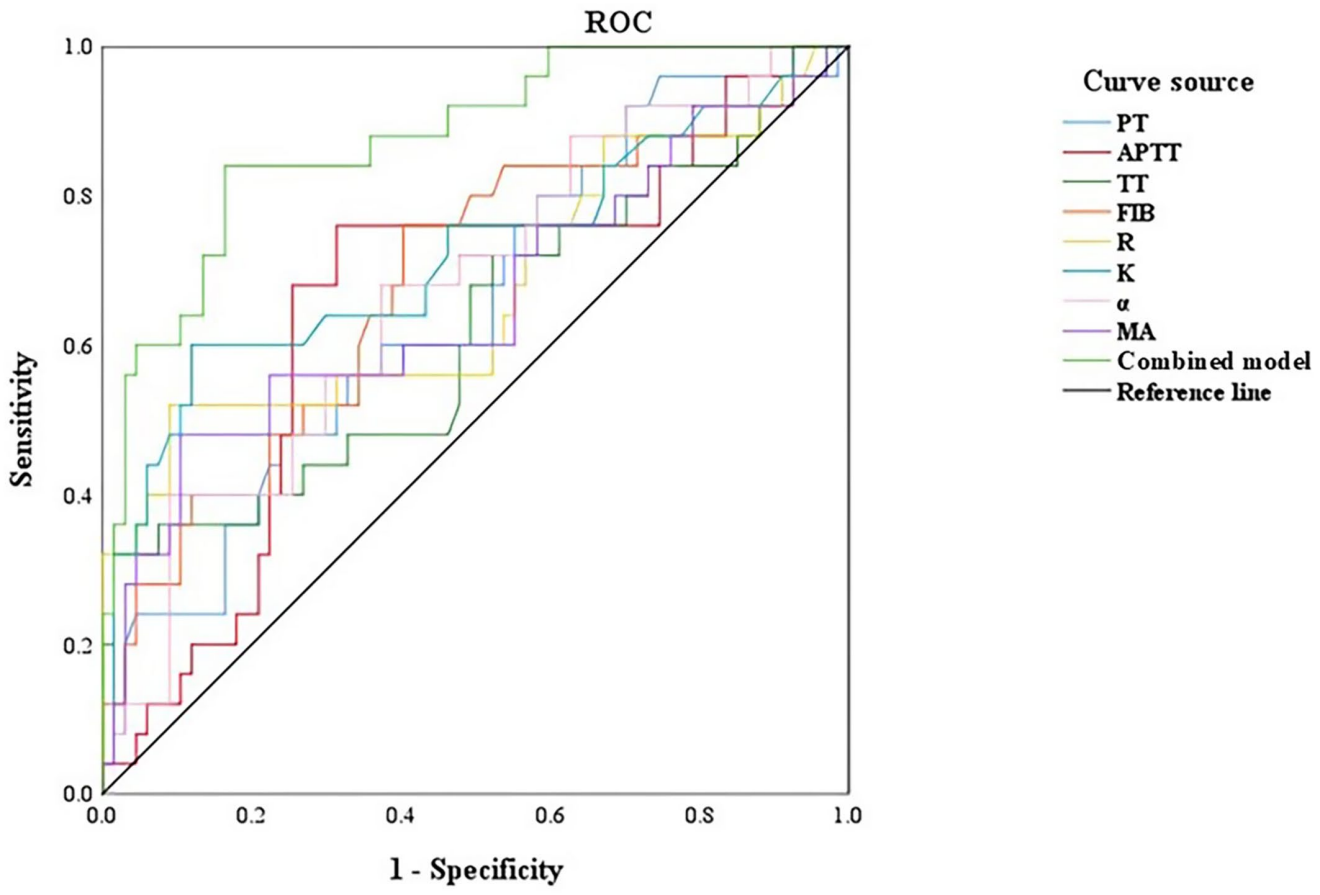
ROC curve of TEG and coagulation function to evaluate the coagulation function of pregnant women in different periods. PT, prothrombin time; APTT, activated partial thromboplastin time; TT, thrombin time; FIB, fibrinogen; R, reaction time; K, kinetics time; α-angle, angle of clot formation; MA, maximum amplitude.

In the first trimester group, the rate of abnormal hypercoagulability was 13.33% (4/30 cases). The rate of abnormal hypercoagulability was 20.59% (7/34 cases) in the second trimester group and 50.00% (14/28 cases) in the third trimester group. There was a significant difference in the rate of abnormal hypercoagulability among pregnant women at different stages of pregnancy (*χ*^2^ = 11.021, *p* < 0.05). A ROC curve was generated to observe and analyze the parameters of TEG and coagulation function with respect to the occurrence of abnormal hypercoagulability. The ROC analysis showed that each single parameter had modest discrimination, whereas the combined model (all eight variables) achieved an AUC of 0.876 with sensitivity 84.00% and specificity 83.60% (Table 4 and Figure 4).

**Table 4:** Value of TEG and coagulation function in evaluating coagulation function of pregnant women in different periods.

Index	AUC	Truncation value	Sensitivity	Specificity degree	Youden index	*p*
PT	0.648	7.55	56.00	67.20	0.232	0.030
APTT	0.655	25.08	76.00	68.70	0.447	0.023
TT	0.619	17.90	32.00	98.50	0.305	0.031
FIB	0.688	3.78	76.87	59.70	0.357	0.006
*R* value	0.673	6.82	52.00	91.00	0.430	0.011
*K* value	0.690	2.78	60.00	88.10	0.481	0.001
α angle	0.670	79.95	40.00	91.00	0.310	0.013
MA value	0.658	72.01	48.00	89.60	0.376	0.020
Combined model	0.876	/	84.00	83.60	0.676	<0.001

PT, prothrombin time; APTT, activated partial thromboplastin time; TT, thrombin time; FIB, fibrinogen; R = reaction time, representing the latency period until initial fibrin formation (min); K = clot formation time, indicating the speed of clot strengthening (min); α-angle = angle between the baseline and the tangent to the clotting curve, reflecting the rate of fibrin build-up (°); MA = maximum amplitude, representing the maximum strength of the formed clot (mm); Youden index = sensitivity + specificity−1; it reflects the overall diagnostic effectiveness of a test, with higher values indicating better discriminative ability.

## 4 Discussion

Pregnancy represents a unique stage for women, during which coagulation status is closely linked to delivery safety. While the hypercoagulable state of pregnant women can facilitate placental exfoliation during delivery, persistent high coagulation levels can elevate the risk of adverse perinatal outcomes such as thrombosis and postpartum hemorrhage (1, 13–15). Therefore, systematic evaluation of coagulation function is beneficial for accurately assessing the coagulation status of pregnant women. It helps identify a prethrombotic state, mitigate pregnancy-related adverse outcomes, and provide reliable guidance for screening strategies. The observed decrease in PT among the routine coagulation indices indicates that the blood is in a hypercoagulable state. PT and APTT are sensitive indicators of the function of factors involved in exogenous and endogenous coagulation pathways (16, 17). TT is the total time required for the conversion of fibrinogen to fibrin, and its shortened duration provides the material basis for the occurrence of a hypercoagulable state in pregnancy (18). FIB is an important factor involved in the coagulation stage, which can reflect whether the body is in a state of hypercoagulability (19). In this study, it was observed that prothrombin time (PT), activated partial thromboplastin time (APTT), and thrombin time (TT) decreased while fibrinogen (FIB) levels increased with advancing gestational age, which aligns with findings reported in previous studies (20). These changes are likely influenced by hormonal fluctuations during pregnancy, which can impact liver metabolism and disrupt the normal function of the coagulation system. It has been documented that beginning from the fifth week of pregnancy, there is a substantial increase in the activity of various coagulation factors (such as V, VII, VIII, IX, etc.) in pregnant women, with activity levels peaking in the third trimester and returning to baseline levels after delivery (21, 22). These findings indicate that pregnant women at different stages of gestation are prone to intravascular coagulation and hypercoagulability due to activation of the coagulation system. However, traditional coagulation function assessments primarily focus on the coagulation system alone, which can be influenced by external factors and may not fully reflect the state of platelets and fibrinolysis within the body. Consequently, these assessments may be inadequate predict the risk of perinatal bleeding or thrombosis in a systematic and accurate manner.

TEG offers a dynamic assessment of the coagulation process by comprehensively evaluating platelet aggregation and fibrin function. It allows clinicians to classify coagulation status (hypo-, normo-, or hypercoagulable) and provides integrated insight into the coagulation cascade and platelet interactions (12, 23, 24). In this study, R and K values decreased, whereas *α*-angle and MA increased with advancing gestational age, indicating a progressive shift toward hypercoagulability throughout pregnancy. These trends are broadly consistent with previous reports. The observed changes in coagulation parameters during first trimester—such as continuous activation of coagulation factors, a rapid increase in platelet count, and accelerated clot formation—may contribute to alterations in fibrinogen levels and, in turn, affect the overall coagulation state of pregnant women. The decrease in R value suggests up-regulated activity of coagulation factors, similar to PT and APTT, reflecting the quality of coagulation factor. The decrease in K value indicates hyperfunction of fibrinogen. An increased MA reflects enhanced platelet function and/or higher fibrinogen levels, whereas a larger *α*-angle indicates faster clot formation and increased fibrinogen activity (25–27). While this study identified associations between TEG parameters and the four traditional blood coagulation tests, it’s important to note that traditional blood coagulation tests assess only plasma components and provide insights into specific stages of the coagulation process, which may have certain limitations. The TEG detection method utilizes whole blood samples, allowing for a comprehensive analysis of both plasma composition and the influence of cellular components on blood coagulation. This approach provides a more holistic view of the blood coagulation process compared to traditional plasma-based tests (28, 29). In this study, the parameters of TEG and coagulation function were analyzed by ROC curve analysis. The hypercoagulable AUC, sensitivity and specificity of the single evaluation of abnormal coagulation function were lower than the joint prediction AUC of 0.876, sensitivity of 84.00% and specificity of 83.60%. Therefore, traditional coagulation function tests and TEG parameters serve distinct purposes and complement each other in screening, diagnosis, and monitoring. Combining both approaches allows for a comprehensive evaluation of blood coagulation function in pregnant women across different stages of pregnancy.

Comparison with prior studies and potential predictive roles. Our trimester-specific trends (↓R, ↓K, ↑*α*, ↑MA) align with reports that global viscoelastic measures shift toward hypercoagulability in third trimester and normalize postpartum (4, 24). In high-risk or bleeding contexts, TEG/ROTEM-guided algorithms have been associated with more targeted transfusion management (5, 8). However, evidence that TEG predicts obstetric complications in healthy populations remains limited and heterogeneous, with device/platform differences and context-specific thresholds complicating synthesis. Prior studies, including work in women with gestational hypertension, preeclampsia and other high-risk obstetric conditions, have suggested that viscoelastic assays such as TEG or ROTEM may detect coagulation abnormalities that are not fully captured by conventional plasma-based tests and may help guide transfusion in bleeding scenarios (30). In contrast to these complication-focused cohorts, our analysis was restricted to strictly defined healthy, low-risk pregnant women, and our primary aim was to characterize physiological, trimester-stratified changes in both TEG and conventional coagulation indices. Within this context we additionally explored a simple combined model incorporating TEG parameters and conventional tests for the laboratory-defined identification of hypercoagulability, rather than to demonstrate overall superiority of TEG or to propose new indications for its use. Existing literature remains heterogeneous with respect to study populations, analytic methods, and decision thresholds, and robust evidence that TEG-based metrics independently predict obstetric complications in healthy women is still limited. On balance, current literature supports contextual, indication-based use of viscoelastic testing rather than universal screening, which is consistent with our interpretation.

However, this study has several limitations. First, this was a retrospective, single-center analysis with a modest sample size (*n* = 92 pregnant women, distributed as 30, 34, and 28 across the first, second, and third trimesters, respectively). This restricts the precision of our estimates, limits statistical power for detailed subgroup analyses, and means that the findings should be interpreted as descriptive of healthy, low-risk women in this setting rather than being directly generalized to all obstetric populations or used to inform population-wide screening policies. Second, the analysis focused on the first, second, and third trimesters without dedicated assessment in the postpartum period, precluding characterization of the trajectory back to the non-pregnant baseline. Third, the study population deliberately excluded women with pregnancy complications such as gestational hypertension and gestational diabetes mellitus, so our results do not apply to high-risk obstetric cohorts in whom coagulation dynamics may differ substantially. Fourth, potentially influential factors—including environmental exposures, lifestyle behaviors, and dietary patterns—were not systematically investigated, which may have introduced unmeasured confounding. Finally, as a retrospective, single-center investigation without longitudinal follow-up within individuals, there is an inherent risk of selection bias, and we were unable to directly assess intra-individual changes in coagulation parameters over time. Taken together, these methodological and scope-related issues highlight the need for prospective, multicenter studies with larger and more diverse populations, serial sampling into the postpartum period, and integration of clinical outcome data to validate and extend our findings.

In this study, trimester-related shifts in conventional plasma-based assays (PT, APTT, TT, FIB) and whole-blood TEG indices were observed, consistent with the physiological hypercoagulable state of pregnancy. While TEG provides a dynamic, global assessment of clot initiation, propagation, and strength, our findings do not support routine, population-level TEG screening in healthy pregnant women. Instead, any clinical use of TEG should be indication-specific and complementary to conventional tests—for example, when rapid whole-blood assessment is needed or when standard assays are inconclusive in defined obstetric contexts. Because the present study did not evaluate clinical outcomes, decision thresholds, or economic endpoints, cost-effectiveness and clinical-impact analyses remain necessary. Future prospective work should (i) predefine obstetric indications and actionable thresholds, (ii) link TEG-guided decisions to maternal–neonatal outcomes, (iii) compare added value over conventional testing using decision-curve or impact analyses, and (iv) establish robust, trimester-specific reference intervals.

In summary, the observed changes in PT, APTT, TT, FIB and TEG parameters across gestation primarily characterize normal physiological adaptation rather than pathology. These results underscore a cautious interpretation: in healthy pregnancies, such patterns should not trigger treatment or imply disease. TEG may serve as a selective adjunct—not a universal screen—when used within clearly defined clinical scenarios and integrated with routine coagulation testing. Larger, prospective studies that incorporate outcome and cost data are required before recommending broader implementation.

## Data Availability

The raw data supporting the conclusions of this article will be made available by the authors, without undue reservation.
