# Correction: Application of thromboelastogram and coagulation function in evaluating coagulation status of pregnant women across different trimesters

**DOI:** 10.3389/fmed.2026.1771150

**Published:** 2026-01-23

**Authors:** Jiayu Li, Jianfen Zhu, Xiaoqian Chen, Mingyu Wang, Ying Sha

**Affiliations:** 1 Clinical Laboratory, Zhangjiagang Second People's Hospital, Zhangjiagang, Jiangsu, China; 2 Department of Obstetrics and Gynecology, Zhangjiagang Second People's Hospital, Zhangjiagang, Jiangsu, China

**Keywords:** coagulation function, perinatal outcomes, pregnancy, thromboelastogram, trimesters

There was a mistake in Figure 1 as published. In the research flow chart, the control group information was incomplete; the label “(2) 30 non-pregnant women” was missing. The corrected Figure 1 appears below.

**Figure 1:**
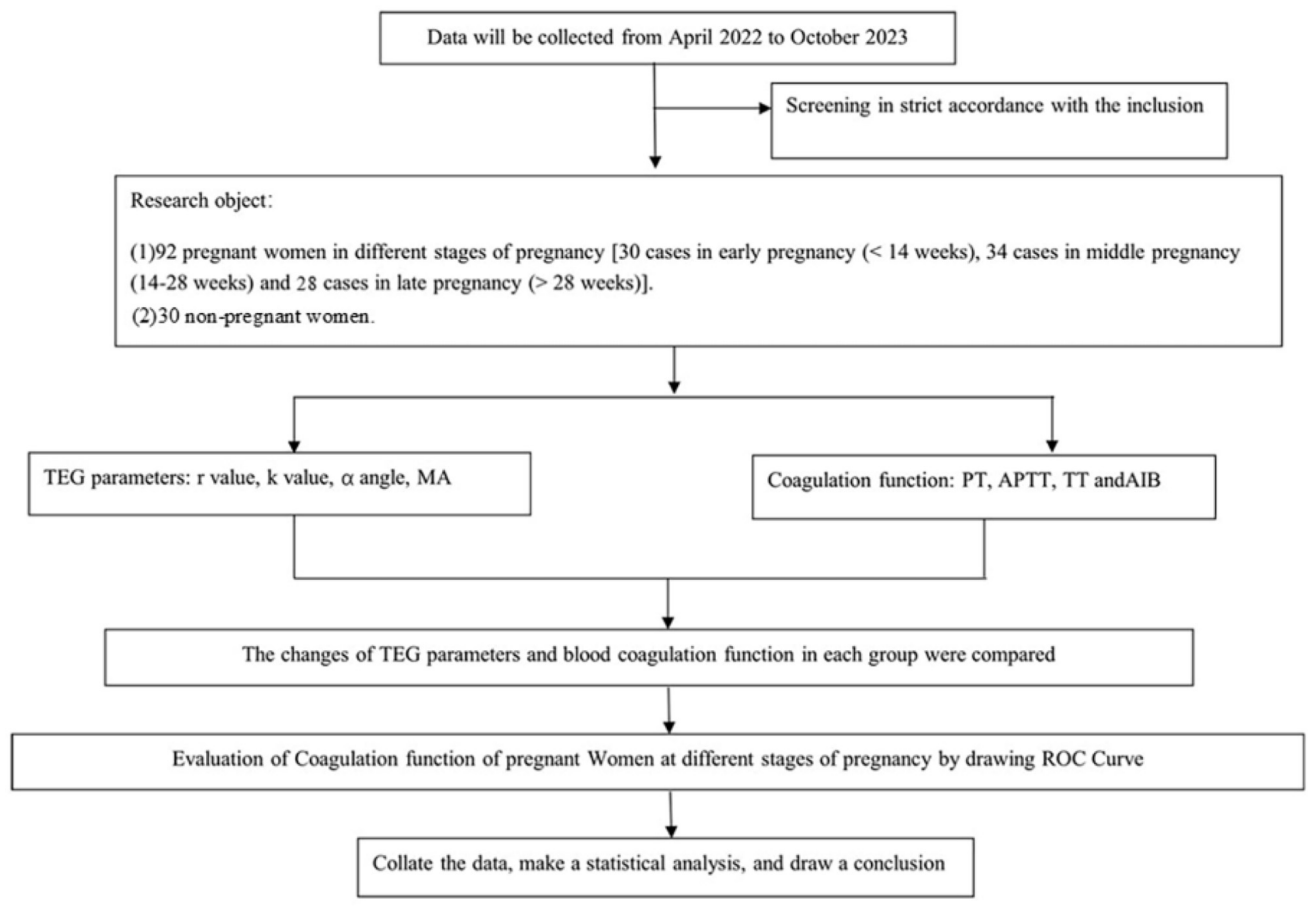
Research flow chart. PT, prothrombin time; APTT, activated partial thromboplastin time; TT, thrombin time; FIB, fibrinogen; TEG, thromboelastography; R, reaction time.

There was a mistake in Figure 2 as published. The group naming in the figure was not updated consistently with the final manuscript terminology. The group labels have been corrected to: Control group, First trimester group, Second trimester group, Third trimester group. In addition, in Figure 2A (PT), the control group value was not synchronized with the corrected values in Table 2; this value has now been updated to match the final table. The corrected Figure 2 appears below.

**Figure 2:**
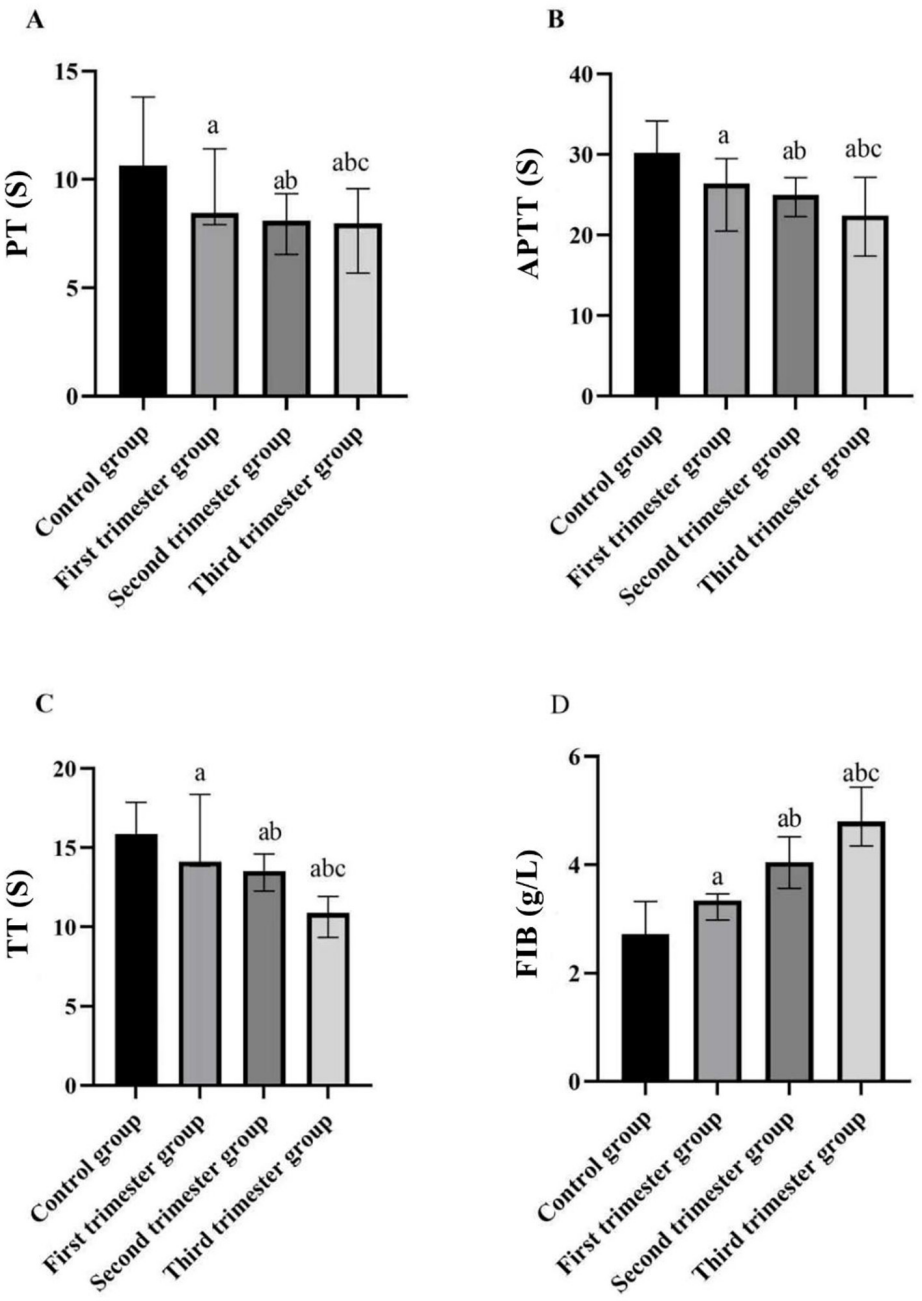
Comparison of four indices of blood coagulation function in different stages of pregnancy and the control group. **(A)** PT (s); **(B)** APTT (s); **(C)** TT (s); **(D)** FIB (g/L) (compared with the control group, ^a^*p* < 0.05; compared with the first trimester group, ^b^*p* < 0.05; compared with the second trimester group, ^c^*p* < 0.05). PT, prothrombin time; APTT, activated partial thromboplastin time; TT, thrombin time; FIB, fibrinogen.

There was a mistake in Figure 3 as published. The group naming in the figure was not updated consistently with the final manuscript terminology. The group labels have been corrected to: Control group, First trimester group, Second trimester group, Third trimester group. The corrected Figure 3 appears below.

**Figure 3:**
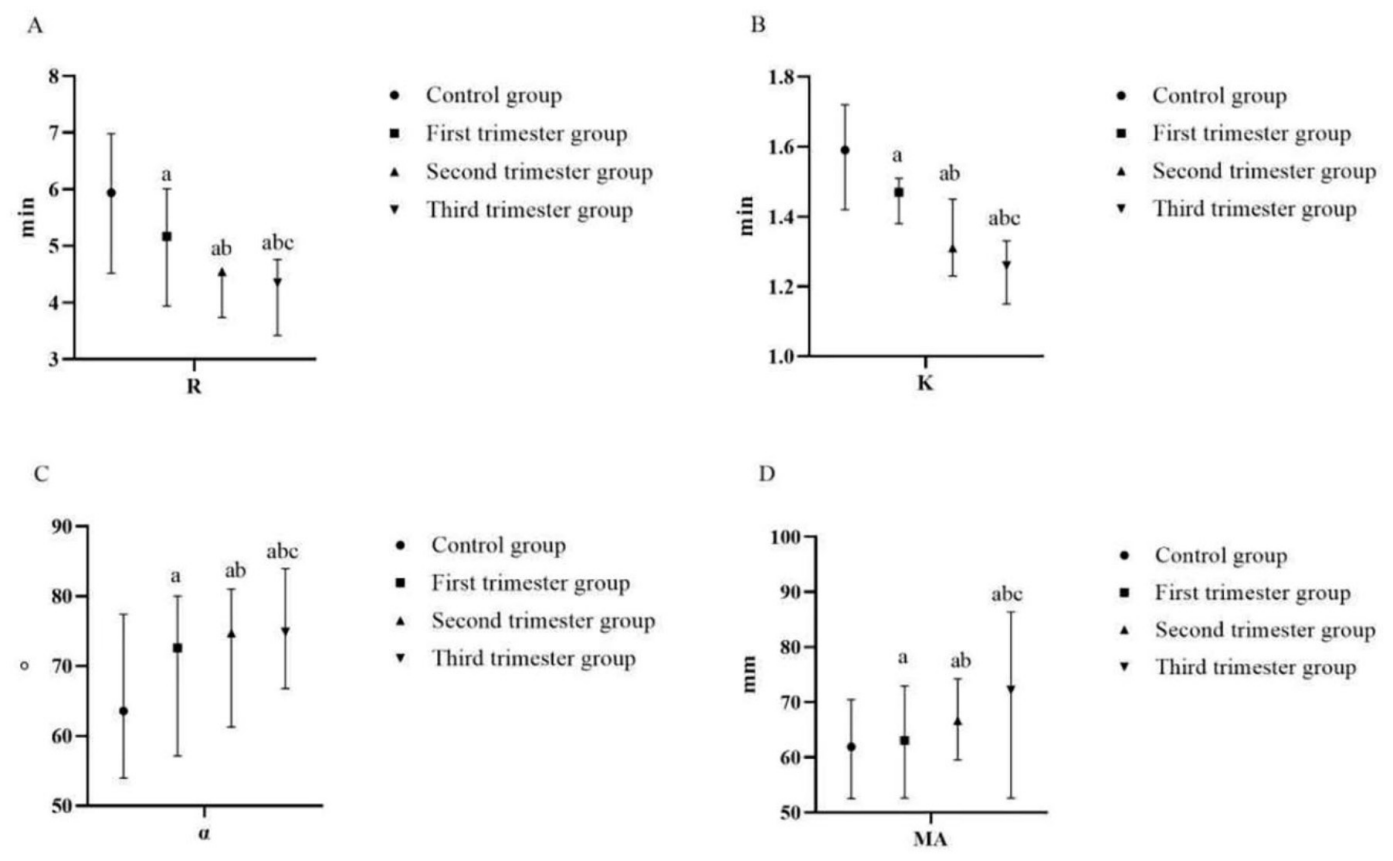
Comparison of TEG of pregnant women at different stages of pregnancy and the control group **(A)** R (min); **(B)** K (min); **(C)** α-angle (°); **(D)** MA (mm) (compared with the control group, ^a^*p* < 0.05; compared with the first trimester group, ^b^*p* < 0.05; compared with the second trimester group, ^c^*p* < 0.05). R, reaction time; K, kinetics time; α-angle, angle of clot formation; MA, maximum amplitude.

There was a mistake in Figure 4 as published. In the legend, the term “United” was used incorrectly and should be corrected to “Combined model” (or “Combined Model”, consistent with the manuscript and Table 4). The corrected Figure 4 appears below.

**Figure 4:**
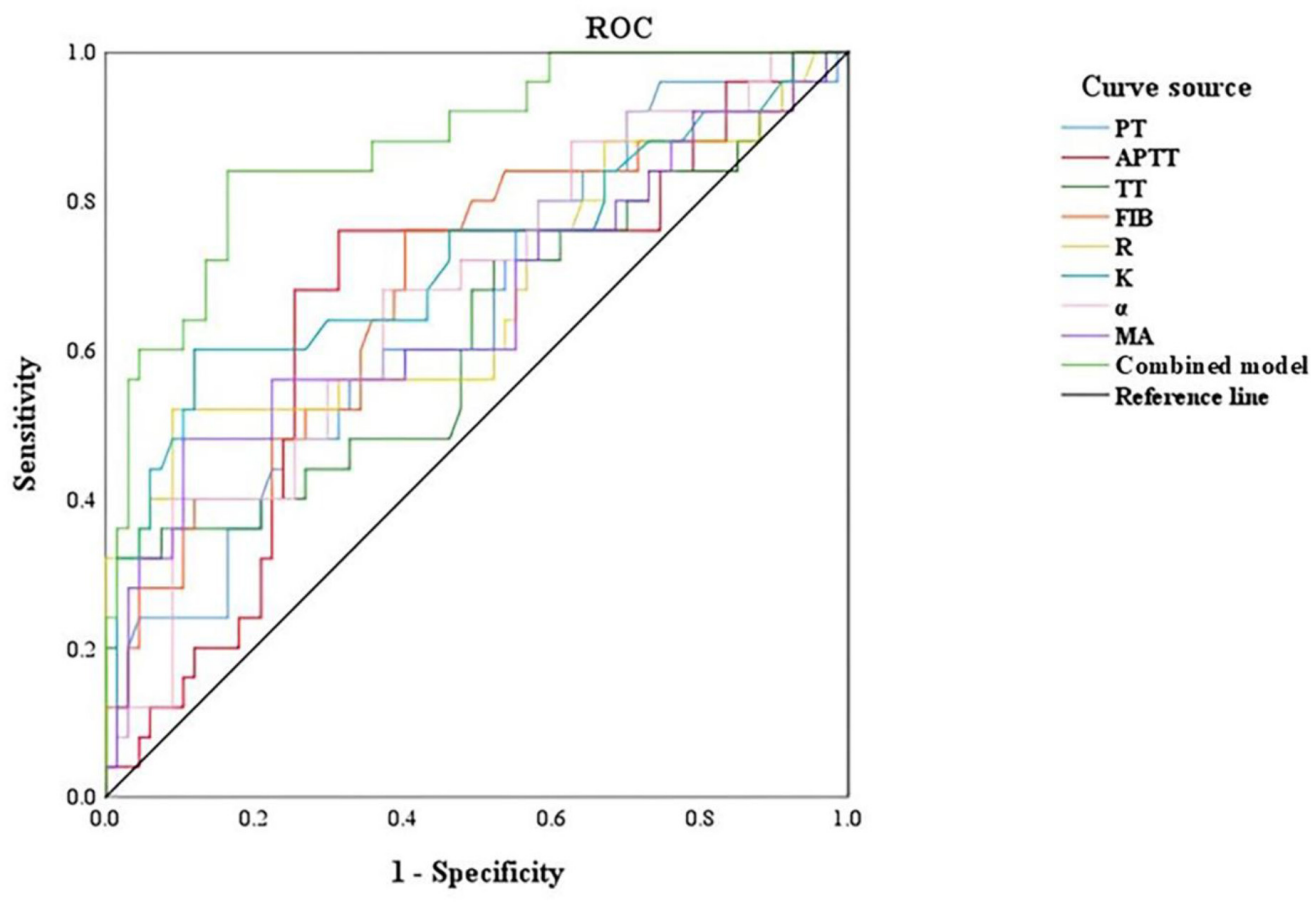
ROC curve of TEG and coagulation function to evaluate the coagulation function of pregnant women in different periods. PT, prothrombin time; APTT, activated partial thromboplastin time; TT, thrombin time; FIB, fibrinogen; R, reaction time; K, kinetics time; α-angle, angle of clot formation; MA, maximum amplitude.

The original version of this article has been updated.

