# Supplementary material for: Application of thromboelastogram and coagulation function in evaluating coagulation status of pregnant women across different trimesters
Source: Front Med (Lausanne). 2025 Dec 12;12:1711912. doi: 10.3389/fmed.2025.1711912 (PMC12741125; doi:10.3389/fmed.2025.1711912)
Supplement: Supplementary file 1 [file Data_Sheet_1.docx]

(1) General data collection

The clinical data of all subjects were obtained from the hospital system, including age, systolic blood pressure, diastolic blood pressure and fasting blood glucose.

(2) Collection of research specimens

All the subjects could eat normally before drawing blood, and the results were not affected by food. Two portions of 2.7mL were collected from vein at routine room temperature and stored in an anticoagulant vacuum tube containing sodium 0.3mL citrate (the proportion was 1:9). The thromboelastography and blood coagulation function should be tested within 4 hours, so as to prevent blood cells from being affected by changes in metabolism, gas and other components.

(3) Traditional blood coagulation test

One sample was centrifuged by 3000r/min and 10min, and detected by automatic blood coagulation analyzer (Wuhan Medical Jie Xun'an Trading Co., Ltd., XL3200C). Prothrombin time (PT), activated partial thromboplastin time (APTT), thrombin time (TT) decreased and fibrinogen (FIB) content was detected by coagulation method. All reagents, quality control products, and calibrations used in this study were purchased from Wuhan Medical Jie Xun'an Trading Co., Ltd. For each project, internal quality control products with high and normal concentrations were prepared. Daily indoor quality control was conducted to ensure that the tests were performed under controlled conditions. Additionally, all test items complied with the interlaboratory quality evaluation standards outlined in the clinical examination guidelines of the Ministry of Health.

(4) TEG detection

One sample was tested by thromboelastographic analyzer [Lepu (Beijing) Medical device Co., Ltd., LEPU-8800]. During the procedure, 1 mL of whole blood anticoagulated with sodium citrate was mixed with 1% sodium citrate solution by gently inverting it five times. After resting for 5 minutes, the sample cup was placed on the detection channel. Using a pipette, 20 μL of 0.2 mmol/L CaCl2 was added to the sample cup, followed by the addition of 340 μL of activated whole blood to avoid introducing bubbles. Then, the sample cup was put into the detection channel for detection, during which the TEG curve and clotting reaction time (R) were recorded: The time required for the first coagulation initiation to the first detected blood clot was 2-8min in the normal range. Blood clot formation time (K): This is the time interval from the end of the R-time to when the elasticity amplitude reaches 20 mm. The normal range is 1 to 3 minutes. Blood clot formation rate (α Angle): This angle is formed between the tangent line and the horizontal line at the maximum curve of the elasticity chart. The normal range is 55 to 78 degrees. Maximum amplitude (MA): This refers to the highest point on the elasticity chart. The normal range is 51 to 75 mm.
